# Systematic mapping review of the factors influencing dietary behaviour in ethnic minority groups living in Europe: a DEDIPAC study

**DOI:** 10.1186/s12966-016-0412-8

**Published:** 2016-07-28

**Authors:** Hibbah Araba Osei-Kwasi, Mary Nicolaou, Katie Powell, Laura Terragni, Lea Maes, Karien Stronks, Nanna Lien, Michelle Holdsworth, on behalf of the DEDIPAC consortium

**Affiliations:** 1 Public Health Section, School of Health and Related Research-ScHARR, The University of Sheffield, Sheffield, UK; 2 Department of Public Health, Academic Medical Centre, University of Amsterdam, Amsterdam, The Netherlands; 3 Department of Nursing and Health Promotion, Faculty of Health Sciences, Oslo and Akershus University College of Applied Sciences, Oslo, Norway; 4 Department of Public Health, Ghent University, Ghent, Belgium; 5 Department of Nutrition, University of Oslo, Oslo, Norway

**Keywords:** Diet, Food habits, Dietary behaviour, Ethnic minority groups, Europe, Migrants, Immigrants, Factors, Determinants

## Abstract

**Background:**

Europe has a growing population of ethnic minority groups whose dietary behaviours are potentially of public health concern. To promote healthier diets, the factors driving dietary behaviours need to be understood. This review mapped the broad range of factors influencing dietary behaviour among ethnic minority groups living in Europe, in order to identify research gaps in the literature to guide future research.

**Methods:**

A systematic mapping review was conducted (protocol registered with PROSPERO 2014: CRD42014013549). Nine databases were searched for quantitative and qualitative primary research published between 1999 and 2014. Ethnic minority groups were defined as immigrants/populations of immigrant background from low and middle income countries, population groups from former Eastern Bloc countries and minority indigenous populations. In synthesizing the findings, all factors were sorted and structured into emerging clusters according to how they were seen to relate to each other.

**Results:**

Thirty-seven of 2965 studies met the inclusion criteria (*n* = 18 quantitative; *n* = 19 qualitative). Most studies were conducted in Northern Europe and were limited to specific European countries, and focused on a selected number of ethnic minority groups, predominantly among populations of South Asian origin. The 63 factors influencing dietary behaviour that emerged were sorted into seven clusters: social and cultural environment (16 factors), food beliefs and perceptions (11 factors), psychosocial (9 factors), social and material resources (5 factors), accessibility of food (10 factors), migration context (7 factors), and the body (5 factors).

**Conclusion:**

This review identified a broad range of factors and clusters influencing dietary behaviour among ethnic minority groups. Gaps in the literature identified a need for researchers to explore the underlying mechanisms that shape dietary behaviours, which can be gleaned from more holistic, systems-based studies exploring relationships between factors and clusters. The dominance of studies exploring ‘differences’ between ethnic minority groups and the majority population in terms of the socio-cultural environment and food beliefs suggests a need for research exploring ‘similarities’. The evidence from this review will feed into developing a framework for the study of factors influencing dietary behaviours in ethnic minority groups in Europe.

**Electronic supplementary material:**

The online version of this article (doi:10.1186/s12966-016-0412-8) contains supplementary material, which is available to authorized users.

## Background

During the last few decades, migration in Europe has increased and many immigrant-origin groups have been reported to have a higher prevalence of diet-related non-communicable diseases (NCDs) and poorer dietary habits than the native born European populations [1, 2]. In addition, Europe has a number of indigenous minority groups such as the Sami and the Roma, who have historically suffered from discrimination and marginalization accompanied by diet-related NCDs [3]. Given the rise in ethnic minority groups and the high prevalence of NCDs among these populations [4], a clear understanding of factors influencing dietary behaviour is warranted in order to assess the needs of these populations and to develop effective public health interventions that also reach ethnic minority groups.

Dietary behaviours have been found to vary widely among and within different ethnic minority groups compared to host populations [5], indicating that factors influencing dietary behaviour may differ in ethnic minority populations as compared to the majority population [6]. However, most studies have focused either on a selected number of ethnic minority groups or are limited to specific European countries [7, 8]. For instance, research in the UK has focused on South Asians [9] and African Caribbeans [7] and in the Netherlands the focus has been on Surinamese [10]. This emphasis is also reflected in reviews of dietary behaviours; a 2008 review focused specifically on dietary change among the largest ethnic minority groups in Europe [11], whilst two reviews concentrated only on ethnic minority groups in the UK [5] and France [12]. Indigenous minorities have not been included in any reviews. Thus there is a lack of insight into the broad range of factors influencing dietary behaviour among a wide range of ethnic minority groups in Europe. In addition, there has been little attempt to study factors influencing dietary behaviour in a holistic way. In the obesity foresight map [13] for instance, the complexity of factors driving dietary behaviours are illustrated, but this was not prepared through the lens of ethnic minority populations.

This review fills these gaps by systematically reviewing primary studies on a wide range of ethnic minority groups and by considering a variety of dietary behaviours over the whole life course, using a holistic and data driven approach, by clustering emerging factors across these groups. The aims of this review were to identify the broad range of factors influencing dietary behaviour among ethnic minority groups living in Europe in order to identify gaps in the literature to guide future research. The evidence from this review will also feed into developing a framework for the study of factors influencing dietary behaviours in ethnic minority groups in Europe [14], as part of the work of the DEDIPAC-KH (DEterminants of DIet and Physical Activity Knowledge Hub) [15] for European populations.

## Review

### Review typology

A systematic mapping review [16] of the factors influencing dietary behaviour among ethnic minority groups in Europe was conducted. A mapping review was selected because it allows the mapping and categorisation of existing literature and identification of the gaps in research literature [16]. To avoid research bias during the review process, the review protocol was registered with PROSPERO (PROSPERO 2014: CRD42014013549) before commencing.

### Search strategy

An initial scoping search was undertaken through PubMed PubReMiner [17] with the aim of assessing the amount of available literature and identifying appropriate search terms to be used in the main searches. A search strategy was constructed in consultation with information specialists from the University of Sheffield and the Academic Medical Centre, Amsterdam. The search strategy was based on search terms within three concepts: (i) dietary behaviours and its synonyms: diet, food habits, nutritional status, food preferences and nutrition; (ii) ethnic minority groups; and (iii) all countries that are listed by the World Bank as low and middle income countries. In addition, countries from the former Eastern European Bloc [18] from where groups commonly migrate to other parts of Europe, were included so that the review captured all ethnic minority groups living in Europe including indigenous populations. Other search terms that were used to capture potential studies were: emigrants, immigrants, cultural diversity, minority groups, migrants, ethnic groups, multiculturalism, ethnic minority, BME (Black and Minority Ethnic), black, minority ethnic, asylum seeker, refugee, non-white, coloured population or black; and (iii) Europe, all European countries by name. The search strategy contained free text and subject headings.

The following nine electronic databases were searched: MEDLINE, EMBASE, Web of Science, Cochrane Library, CINAHL, ProQuest, Psychinfo, ASSIA, and Campbell Collaboration Library of Systematic Reviews. The search strategy was modified where necessary for use in different electronic databases. The complete MEDLINE search strategy is shown in Additional file 1.

Databases were searched from 1999 to 2014, as it was expected that any factor identified before 1999 would also be referred to in more recent literature. Spot checks on results from the scoping review indicated that key papers emerged after 1999. Searches were conducted between May and July 2014. The citation follow–up technique and contacting of experts in the field was undertaken to identify additional relevant articles. In addition, the reference lists of all included articles were scanned for articles that met the inclusion criteria. All citations were downloaded into an Endnote web library and duplicates were removed.

Ethnic minority population is a concept used for very heterogeneous groups that may share minority status in their country of residence due to ethnicity, place of birth, language, religion, citizenship as well as other cultural differences [19]. This definition may include groups from newly arrived immigrants to (minority) groups that have been part of a country’s history, for instance the Sami people.

### Inclusion and exclusion criteria

Observational and intervention studies, using quantitative, qualitative or mixed methods that examine dietary behaviour among ethnic minority groups in Europe were included. Other studies that focused on nutrition-related conditions, for example, obesity and contained relevant data on dietary behaviour in ethnic minority groups were also included. All studies that identified an association between a factor (including correlates, predictors, moderators, determinants and mediators) and dietary behaviour of minority groups living in Europe were retained.

All primary studies that analysed diet as a confounder in a relationship between ethnicity and disease were excluded. As were studies that explored whether ethnicity is a determinant of diet and did not attempt to explain why, non-human studies/laboratory based studies and studies examining beliefs and practices around breastfeeding and weaning. Studies examining the nutrient status of particular groups without mention of diet and studies presenting descriptive information about diet were also excluded.

### Study selection

The title and abstracts of a total of 2965 articles identified references were imported into Endnote and 730 duplicates removed. The remaining 2235 articles were equally divided between five independent reviewers (HO, MN, KP, MH and LT) against the inclusion criteria. Of these abstracts, 1956 articles did not meet the inclusion criteria. The main reasons for excluding studies were because they contained no empirical data on ethnic minority groups, presented only descriptive information on diet or where outside the review time limit. Full text articles were retrieved by the five reviewers for the remaining 279 articles and the inclusion/exclusion criteria were applied. This yielded 68 potentially relevant papers for data extraction. Spot checks were conducted on a sample of 10 % of the excluded papers to assess the extent of agreement between reviewers. During the spot checks, there was a good degree of concordance. There was disagreement between two reviewers on two papers, therefore a third reviewer in the team was consulted. The outcome in both cases was to exclude the papers. The most common reason for excluding studies from this review during the data extraction stage was because they were focused on describing dietary differences between populations without examining the factors driving dietary behaviour.

**Fig. 1:**
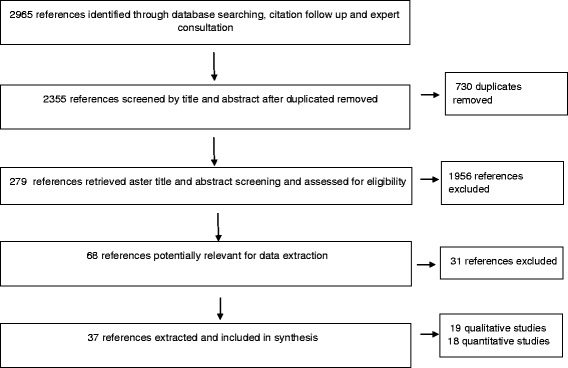
PRISMA flow diagram of systematic mapping search and selection process

During the data extraction process 37 studies met the inclusion criteria (Fig. 1).

### Data extraction and synthesis

Data extraction was performed by the five reviewers according to the following study characteristics: study design, sampling population, sample characteristics, number of participants, country where the study was conducted, sampling method, dietary behaviour measured, factors reported to influence dietary behaviour. As the main aim of this mapping review was to gather a broad base of evidence to map the conceptual domain of factors potentially influencing dietary behaviours it was decided to include all factors reported by authors and not to restrict to those factors where a statistical relationship had been demonstrated. For quantitative studies, types of analysis were also extracted, whilst direct quotes were extracted from qualitative studies.

### Quality assessment

Quality assessment of quantitative and qualitative studies was undertaken using the standard quality assessment criteria for evaluating primary research papers [20]. Each reviewer in the process of extracting data assessed the paper for quality as well. To ensure consistency in quality assessment, an independent reviewer (LM) cross-checked the quality assessment between the five reviewers. Additional files (Additional files 2 and Additional file 3) summarise the quality assessment scores given to the included studies.

### Data analysis and emerging clusters

Two stages were used in the analysis. In the first stage, all factors influencing dietary behaviour identified in the selected papers were extracted and their association described with the different dietary behaviours observed.

The second stage involved sorting and structuring the factors into clusters, during which, the list of factors emerging from the review were clustered according to how they were seen to relate to each other, in a data driven approach [21]. This clustering step was part of a larger concept mapping process leading to the development of a systems-based framework of factors influencing dietary behaviour and physical activity/sedentary behaviour in ethnic minority groups living in Europe. The use of the concept mapping methodology [21] is explored in detail in a separate paper [14]. The concept mapping approach is guided by systems thinking, which can simply be defined as “looking at things in terms of the bigger picture” [22]. This concept mapping approach included steps on generating a list of factors, and sorting and structuring these factors into clusters by consensus based on how they relate to each other [19].

Within the DEDIPAC-KH, an inter-disciplinary group focused on the determinants of dietary and physical activity behaviours. This comprised a team specifically focussing on the factors influencing the behaviours of ethnic minority populations (‘DEDIPAC ethnic minority team’). Other parts of the DEDIPAC-KH focussed on the factors influencing dietary [23, 24] (Condello G, Ling FCM, Bianco A, Chastin S, Cardon G, Ciarapica D, et al. Using Concept Mapping in the Development of the EU-PAD Framework (European Physical Activity Determinants across the Life Course): a DEDIPAC-Study. Submitted) and physical activity behaviours of the general European population (‘DEDIPAC general population team’).

All 63 factors were grouped into seven clusters (Table 1) in two steps - firstly by a meeting of *n* = 5 members of the DEDIPAC ethnic minority team, followed by a confirmatory workshop of *n* = 21 members of DEDIPAC general population team. Experts representing a range of disciplines participated in the clustering process: nutrition, public health, epidemiology, anthropology, social demography, economics, sociology, dietetics, psychology, exercise physiology, health promotion and physical activity.

**Table 1:** Dietary map of the 63 factors and the 7 clusters that emerged from the systematic mapping review

	Migration context	Social and cultural environment	Food beliefs and perceptions	Accessibility of food	The body	Psychosocial	Social and material resources
Number of factors	7	16	11	10	5	9	5
Factors	Region of origin Urban or rural dweller Age at migration Country of birth Length of stay in host country Place of residence in host country Westernization	Cultural identity Ethnic identity Ethnicity Religious beliefs Equipping children in different social networks Perception of host culture Level of acculturation Religious prescriptions Socialization process in place of residence Conformity to tradition Traditional dietary values/beliefs Gender Age Social networks Social ties Social bonding	Status of traditional vs convenience foods/diets Familiarization of host foods before migration Familiarization with host country foods Husband's food preferences Children's food preferences Inter-generational influences on diet Parental dietary habits Perception of healthy foods Food beliefs Perception of cost Social role of food	Availability of traditional foods Accessibility of traditional foods Food prices Food-related life-style Neighbourhood level physical proximity Season Family’s neighbourhood (ethnic enclave) Lack of time for cooking traditional foods Time for food preparation Change in lifestyle (work/school commitments)	Health consciousness Dieting Tendency BMI Body image perception and preferences for larger body size Child’s health	Taste preferences Attitudes Subjective norms Perceived behaviour control Perceived behavioural intention Perceived group norms Past behaviour Motivation Food neophobia	Competency in host language Educational attainment SES Income Nutrition knowledge

## Results

### Description of included studies

The characteristics of the 37 included studies are presented in Tables 2 and 3 (19 qualitative; 18 quantitative). Most of the studies were conducted in Northern Europe, i.e.,(UK *n* = 9, Norway *n* = 9, The Netherlands *n* = 6 and Sweden *n* = 5). The most commonly studied ethnic minority groups were Pakistanis and Bangladeshis. Most of the studies were conducted among adults (*n* = 31), but studies on children/adolescents (*n* = 5) and on older adults (*n* = 1) were found. However, 11 of the studies conducted on adults included participants who were older adults and 3 other studies included children/adolescents. The number of participants ranged from 14 to 83 in the qualitative studies and 100–12,811 in the quantitative studies.

**Table 2:** Characteristics of quantitative studies on factors influencing dietary behaviour in minority groups

Author	Country	Study population	Design	Participants	Dietary behaviour measured
Koochek et al., 2001 [27]	Sweden/Iran	Iranian-born residents of Stockholm/Iranians living in Iran	Cross- -sectional	Elderly ≥60 year Iranians in Sweden (*N* = 121; *F* = 66 %) Iranians in Teheran (*N* = 52; F = 40 %)	Dietary intake including fruit and vegetables
Volken et al., 2013 [28]	Switzerland	Portuguese, German, Italian, Turkish, Serbian, Kosovan residents of Switzerland.	Cross-sectional	Participants aged 17–74 years. M = 5390, F = 6358	Fruit and vegetable intake
Edwards et al., 2010 [39]	UK	36 nationalities of international students	Cross-sectional	Participant aged 20–60 year. *N* = 226 M = 31 %, F = 69 %	Food neophobia, changes in eating habit
Ross et al., 2009 [59]	Sweden	Sami involved in reindeer herding (traditional lifestyle) vs others	Cross-sectional	*N* = 595 Sami (F = 321, M = 274)	Food and nutrient intake
Skreblin, et al., 2003 [29].	Croatia	Three groups of adolescents: host; immigrant (Bosnia Herzegovina); permanently settled	Cross-sectional	*N* = 510 adolescents (14–19 years.)	Food intake, dieting practice
Brustad et al., 2007 [47]	Norway	SAMI and Norwegian who had childhood in SAMINOR study	Cross-sectional	Participant aged 36–79 years. *N* = 7614 both M & F	Dietary patterns in childhood based on clustering of 11 ‘traditional’ Sami food items
Brustad et al., 2008 [60]	Norway	SAMI and Norwegian	Cross-sectional	Participant aged 36–79 years. *N* = 12811 Age: both M & F	Dietary patterns based on traditional and modern dietary items.
Kumar et al., 2004 [26]	Norway	East Asians, Indians, sub-Saharan Africa, Middle East/North Africa-	Cross-sectional	Adolescents resident in Oslo. Mean age 15.6 years.; *N* = 1659 (M = 48.9 %,F = 51.1 %)	Fruit and vegetable intake, breakfast skipping.
Kassam-Khamis et al., 2000 [9]	UK	South Asian Muslims from Bangladesh, Pakistan East Africa (Ismailis)	Cross-sectional	Households include everyone >12 years. *N* = 291 individuals in 92 households (*n* = 100 Bangladeshis; *n* = 108 Pakistanis; *n* = 83 Ismailis)	Food intake
Harding et al., 2008 [61]	UK	Black Caribbean, Black African, Pakistani, Indian Bangladeshi	Cross-sectional	Children aged 11–13 years. pupils in 51 schools *N* = 6599	Food intake including fizzy drinks, fruit and vegetables, breakfast
Nicolaou et al., 2006 [10]	Netherlands	Surinamese of South Asian and African origin, white Dutch	Cross-sectional	Adults aged 35–60 year. *N* = 1518	“Diet Quality” based on the Intake of a number of key foods and breakfast
Nielsen et al., 2014 [50]	Denmark	Non Western minorities; Turkish (35 %) Pakistani/Indian background (20 %), “other” covers >100 different countries	Cross-sectional	Parents with children 6 months to 3.5 years. Danish and non-western *N* = 337	Dietary intake, dietary pattern (healthy eating)
Carrus et al., 2009 [49]	Italy	Indian females	Cross-sectional	Females 18–34 years. living in Rome for ≥ 10 year *N* = 100	Purchase of ethnic food
Perez-Cueto 2009 [40]	Belgium	International students from 60 nationalities	Cross-sectional	Students aged 19–48 years. *N* = 235; M = 54 %	Perceived changes in dietary habits, healthy intake
Kjøllesdal et al., 2013 [62]	Norway	Pakistani women with type 2 diabetes	RCT	Participant aged 25–62 years. *N* = 198	Change in food intake
Kjollesdal et al., 2010 [63]	Norway	Pakistani women living in Norway and born in Pakistan or born in Norway by two Pakistani parents.	RCT	Women aged 28–62 years. *N* = 82	Healthy dietary intake
Khunti et al., 2008 [64]	UK	Schools with a >60 % South Asian population, mainly Indian origin.	Action research	Pupils aged 11–15 years, *N* = 4763, (77 % South Asian)	Dietary pattern (healthy and unhealthy intake)
Johansen et al., 2010 [65]	Norway	Women living in Norway and born in Pakistan or women born in Norway by two Pakistani parents.	RCT	Women aged 25–63 years. *N* = 198	Dietary intake, portion size, intention to change diet

**Table 3:** Characteristics of qualitative studies on factors influencing dietary behaviour in minority groups

Author	Country	Study population	Design	Participants	Dietary behaviour measured
Lawrence et al., 2007 [43]	UK	African (Somalia, Zimbabwe) South Asian (Pakistani/Bangladeshi) females	6 Focus groups	Girls and young women aged 12–35 years *N* = 33	Food choice
Lawton et al., 2008 [36]	UK	Pakistanis, Indians with type 2 diabetes	In-depth interviews	Adults aged 33–71 year. M = 15, F = 17 *N* = 32	Food and eating practices, dietary change
Fargerli et al., 2005 [25]	Norway	Pakistani-born living in Oslo	In-depth interviews	Adults aged 38–66 years. M = 4, F = 11 *N* = 15	Changes in food -habits whilst living in Norway after diabetes diagnosis
Garnweidner et al., 2012 [41]	Norway	Female immigrants form 11 African and Asian countries residing in Oslo	In-depth interviews	Participants aged 25–60 year. *N* = 21	Food habits, meal preparation, perception of change in food habits
Halkier et al., 2011 [66]	Denmark	Pakistani living in Denmark	Interviews, participant observation	*N* = 19 Age = 15–65 years.	Healthy eating practices
Kohinor et al., 2011 [30]	Netherlands	Dutch Surinamese	Semi-structured interviews	*N* = 32 M = 12, F = 20	Healthy dietary intake
Ahlqvist et al., 2000 [67]	Sweden	Iranian women living in Sweden	Interviews	Women aged 29–85 years. *N* = 14	Food intake
Grace et al., 2008 [38]	UK	Bangladeshi adults	17 focus groups and 8 interviews	Bangladeshis without diabetes (M = 37; F = 43); religious leaders (M = 14, F = 15); health professionals (F = 19; M = 1)	Dietary intake in relation to the prevention of type 2 diabetes
Terrangi et al., 2014 [42]	Norway	Somali, Pakistani,Sri Lanka, Iraq, Turkey, Iran, Egypt, Algeria, Lebanon, Morocco	semi-structured interviews	Women aged 25–70 year. *N* = 21	Shopping, preparation and eating habits, dietary acculturation
Jonsson et al., 2002 [31]	Sweden	Somalians	Focus group interviews	19 women with children <18 years.	Food choice, tradition, meanings attached to ‘feeding the family’
Hendriks et al., 2012 [32]	Netherlands	Surinamese Indians	Semi-structured interviews and focus groups	Participants aged 29–83 years. F = 24. M = 3 *N* = 27	Eating habits
Rawlins et al., 2013 [37]	UK	African; Caribbean; Indian, Pakistani, Bangladeshi	Focus groups and interviews	Children aged 8–13 years. and their parents *N* = 43 parents, *N* = 70 children	Perception of healthy eating and shopping practices
Tuomaimen 2009 [33]	UK	Ghanaians	Indepth-interview and participant observation	18 households (*N* = 41 individuals), 24 key informants	Meal format, eating pattern, meal cycle, shopping practices, food preferences
Nicolaou et al., 2009 [34]	Netherlands	Turkish/Moroccan	14 Focus groups	*N* = 83 aged = 20–40 year.	Food intake
Nicolaou et al., 2013 [8]	Netherlands	South Asian Surinamese	Focus group discussions	*N* = 5 Adults (*N* = 4-6 per group);	Food intake, healthy eating
Nicolaou et al., 2012 [44]	Netherlands Morocco	Moroccan	8 focus groups	*N* = 53 aged = 16–59 years.	Changes in and diet
Nielsen 2013 [48]	Denmark	Turkish and Pakistani mothers living in Denmark	Focus groups	Mothers aged = 25–35 years with at least one child < 30 months *N* = 20	Food choice, eating behaviour
Jonsson 2002 [35]	Sweden	Bosnian Muslim immigrants in Sweden.	Focus groups	*N* = 20 Women with children <18 years.	Food choice
Mellin-Olsen et al., 2005 [68]	Norway	Pakistani immigrants in Norway	Focus groups	*N* = 25 women,	Dietary change in meal pattern, meal preparation, intake of specific foods

The 12 dietary behaviours (as stated by authors) examined in the included studies (Tables 2 and 3) were food intake, fruit and vegetable intake, changes in food habits, intention to change diet, product purchase, meal preparation, dietary acculturation, dietary intake (healthy/unhealthy intake), eating habits, food neophobia, dieting and diet quality. Dietary behaviour as used in this review encompasses all food related behaviours that are grouped into three main categories [23] 1. Food choice, consists of outcomes preceding the actual consumption (e.g., produce purchase and intentions); 2. Eating behaviour, comprising outcomes to do with the actual act of eating (e.g., dieting and food neophobia); and 3. Dietary intake consisting of all outcomes related to what is consumed (e.g., fruits and vegetable intake and healthy versus unhealthy.

### Factors influencing dietary behaviours

There were some commonalities and differences in factors influencing dietary behaviour across different ethnic minority groups. For instance, lack of availability of traditional food, foods meeting religious prescriptions, and preferred foods were identified in all study populations irrespective of the region of origin or country of settlement. Religious beliefs and prescription was a common factor influencing dietary behaviour in all studies conducted among South Asians (Pakistanis, Bangladeshis, Indians), African, Middle Eastern and Eastern European immigrants. Taste preference was another frequently reported factor, irrespective of the population or setting of the study. Factors reported by specific populations included fluency in the host language identified in a study conducted among diabetic Pakistani born persons living in Oslo [25] and traditional lifestyle as deduced from occupation, e.g., reindeer herder reported in both papers addressing diet among the Sami and non-Sami groups in Norway (41, 47). Also a number of studies found differential associations between socioeconomic status (SES) and dietary behaviour [9, 10, 26–29]. For instance, SES was not associated with dietary behaviour among populations from East Asia, Indians and African origin in Norway [26], or in a Surinamese population in the Netherlands [10], whilst in a study conducted in the UK, SES influenced dietary intake among South Asians from Bangladesh. All factors are shown in Table 4.

**Table 4:** Emerging factors and their association with dietary behaviours across different populations

Cluster	Factor	Dietary behaviour	Evidence	Study population
Migration context	Region of origin	Eating behaviour	[33]	Ghanaians
	Urban or rural dweller	Food neophobia	[39]	International students
		Fruit and vegetable intake	[28]	Portuguese, German, Italian, Turkish, Serbian, Kosovan residents of Switzerland
	Country of birth	Food neophobia	[39]	International students
	Length of stay in host country	Food neophobia	[29, 39]	International students; Immigrants from Bosnia and Herzegovina
	Place of residence in host country	Food intake	[29]	Immigrants from Bosnia and Herzegovina
	Age at migration	Diet quality	[10]	Surinamese of South Asian and African origin
	Westernization	Changes in diet	[38]	Moroccans
Social and cultural environment	Cultural identity	Food intake	[9, 34]	South Asian, East Africa (Ismailis), Somalian
		Food choice	[31]	Turkish/Moroccan
		Changes in diet	[44]	Moroccan
		Healthy dietary intake	[30]	Dutch Surinamese
		Eating habits	[32]	Surinamese Indians
		Eating behaviour and food choice	[33]	Ghanaians
	Religious beliefs	Food choice	[35]	Bosnian Muslim immigrants in Sweden
		Eating behaviour and dietary change	[36]	South Asians with type 2 diabetes
		Perception of healthy eating and shopping practices	[37]	African Caribbean and South Asian
		Dietary intake in relation to type 2 diabetes	[69]	South Asian
	Perception of host culture	Eating behaviour, meal preparation, perception of change in food habits	[41]	African and Asian
	Level of acculturation	Food intake	[29, 34]	Turkish/Moroccan; Immigrants from Bosnia and Herzegovina
	Religious prescriptions	Shopping, preparation and eating behaviour, dietary acculturation	[42]	Somali, Pakistani, Sri Lanka, Iraq, Turkey, Iran, Egypt, Algeria, Lebanon, Morocco
	Socialization process in place of residence	Food intake	[29]	Immigrants from Bosnia and Herzegovina
	Conformity to tradition	Food choice	[31, 33]	Somalians, Ghanaians
	Traditional dietary values/beliefs	Perception of healthy eating and shopping practices	[37]	African Caribbean and South Asian
	Gender	Fruit and vegetable intake	[28]	Portuguese, German, Italian, Turkish, Serbian, Kosovan residents of Switzerland.
		Food neophobia	[39]	International students
		Dietary intake	[47]	Sami
	Social networks	Changes in diet	[68]	South Asian
	Social ties	Food intake	[34]	Turkish/Moroccan
	Age	Dietary intake	[27]	Iranian
		Fruit and vegetable intake	[28]	Portuguese, German, Italian, Turkish, Serbian, Kosovan residents of Switzerland
	Social bonding	Eating behaviour and dietary change	[36]	South Asians with type 2 diabetes
	Taste preferences	Healthy dietary intake	[41, 66]	African and Asian; South Asian
		Food choice	[30]	Dutch Surinamese
		Dietary intake (healthy/unhealthy intake)	[31]	Somalian
		Food habits, meal preparation	[64]	South Asian
Food beliefs and perceptions	Status of traditional vs convenience foods/diets	Healthy dietary intake	[66]	South Asian
	Familiarization of host foods before migration	Eating behaviour and food choice	[33]	Ghanaians
	Familiarization with host country foods	Food choice	[35]	Bosnian Muslim immigrants in Sweden
	Husband's food preferences	Changes in diet	[44]	Moroccan
	Children's food preferences	Changes in diet	[44, 68]	Moroccan; South Asian
	Inter-generational influences on diet	Food intake	[67]	Iranian
	Parental dietary habits	Food intake	[29]	Immigrants from Bosnia and Herzegovina
	Perception of healthy foods	Healthy dietary intake	[40]	International students
	Food beliefs	Healthy dietary intake	[30]	Dutch Surinamese
		Food intake	[67]	Iranian
		Perception of healthy eating and shopping practices	[37]	African Caribbean and South Asian
	Perception of cost	Perception of healthy eating and shopping practices	[37]	African Caribbean and South Asian
		Dietary intake (healthy and unhealthy intake)	[64]	South Asian
		Food choice	[43]	African, South Asian
	Social role of food	Healthy dietary intake	[63]	South Asian
Accessibility of food	Availability of traditional foods	Food intake; Food choice	[9, 43]	South Asian, East Africa (Ismailis); African south Asian
	Food prices	Perception of healthy eating and shopping practices	[37]	African Caribbean and South Asian
		Food choice	[43]	African, South Asian
	Neighbourhood level physical proximity	Perception of healthy eating and shopping practices	[37]	African Caribbean, South Asian
	Accessibility	Dietary intake	[38]	South Asian
		Changes in diet	[68]	South Asian
	Season	Food intake	[9]	South Asian, East Africa (Ismailis)
		Changes in diet	[68]	South Asian
	Food-related life-style	Shopping, preparation and eating habits, dietary acculturation	[42]	Somali, Pakistani, Sri Lanka, Iraq, Turkey, Iran, Egypt, Algeria, Lebanon, Morocco
	Lack of time for cooking traditional foods	Food habits, meal preparation	[41]	African and Asian
		Healthy dietary intake	[66]	South Asian
	Time for food preparation	Dietary intake (healthy and unhealthy intake)	[64]	South Asian
		Food choice	[43]	African, South Asian
	Change in lifestyle (work/school commitments)	Changes in diet	[44]	Moroccan
		Food choice	[31]	Somalian
		Food intake	[34]	Turkish/Moroccan
		Changes in diet	[44]	Moroccan
The body	Health consciousness	Food choice	[43]	African, south Asian
		Changes in diet	[68]	South Asian
	Dieting Tendency	Breakfast skipping	[26]	East Asians, Indians, sub-Saharan Africa, Middle East/North Africa
	Body image perception and preferences for larger body size	Dieting practice	[29]	Immigrants from Bosnia and Herzegovina
	Child’s health	Food choice	[48]	Turkish and Pakistani mothers
Psychosocial	Taste preferences	Dietary intake (healthy and unhealthy intake)	[64]	South Asian
		Food habits, meal preparation	[41]	African and Asian
		Healthy dietary intake	[66] [30]	South Asian, Dutch Surinamese
		Food choice	[31]	Somalian
	Attitudes	Purchase of ethnic food	[49]	South Asian
	Subjective norms	Purchase of ethnic food	[49]	South Asian
	Perceived behavioural intention	Purchase of ethnic food	[49]	South Asian
	Perceived group norms	Purchase of ethnic food	[49]	South Asian
	Past behaviour	Purchase of ethnic food	[49]	South Asian
	Motivation	Dietary intake (healthy and unhealthy intake)	[64]	South Asian
Social and material resources	Competency in host language	Changes in diet; Dietary intake	[25, 38]	South Asian
	Educational attainment	Dietary intake	[60]	Sami
	SES	Food intake	[9, 28, 29]	Immigrants from Bosnia Herzegovina; Portuguese, German, Italian, Turkish, Serbian, Kosovan residents of Switzerland; South Asian Muslims from Bangladesh, Pakistan East Africa (Ismailis)
	Income	Diet quality	[10]	Surinamese of South Asian and African origin
	Nutrition knowledge	Dietary intake	[38]	South Asian
		Changes in diet	[62]	South Asian

### Emerging clusters

Sixty-three individual factors resulted from the first step of the analysis and seven clusters emerged from the brainstorming and structuring process: social and cultural environment (16 factors), food beliefs and perceptions (11 factors), psychosocial (9 factors), accessibility of food (10 factors), social and material resources (5 factors), migration context (7 factors), and the body (5 factors). As shown in Table 1, the ‘social and cultural environment’ cluster contained the highest number of factors influencing dietary behaviours. These factors include cultural identity and desire to maintain traditional food identity [9, 30–34], religious beliefs and prescriptions [25, 35–38], social networks [10], social bonding [36], level of acculturation and socialization processes [10, 29, 34], social norms/social role of food [38] and gender [28, 39, 40]. Another set of factors was grouped under the cluster ‘accessibility of food’. This cluster includes factors relating to: availability of food in new environments and workplaces and included specific foods, such as traditional, ‘halal’, healthy or preferred foods [25, 35–38, 40–42], accessibility of food (e.g., physical access to traditional foods) [33, 37, 38, 43] and food price [37, 43]. Several factors were also grouped in the ‘food beliefs and perceptions’ cluster: beliefs regarding traditional foods and convenience foods [9, 31], family member preferences (husband/children) [31, 41, 44, 45], parental dietary habits [29], familiarisation of host foods before migration [33], familiarisation of food in new environments [35] and new ways of shopping [42], beliefs and perceptions of healthy food [30, 37, 43], and perception of cost [37]. The ‘migration context’ cluster consists of factors influencing dietary behaviour such as region of origin [33, 46] and country of origin [29, 47] length of stay [29, 39] and age [10]. The ‘body’ cluster includes factors such as health [35, 38, 48], dieting [26], BMI [27] and body size preferences [23]. The ‘psychosocial’ cluster included factors such perceived behavioural control, perceived grouped norms [49, 50], taste preference [41], motivation [50] and past behaviours [49]. This cluster of factors seems relevant to the South Asian population as shown in Table 4. The last set of factors was grouped under the ‘social and material resources’ cluster, which includes education [27], SES (index of income and education) [9, 10, 37, 49], competency in host language [25, 38], nutritional knowledge [35, 38], change in lifestyle (lifestyle referring to work/school commitments) and time for food preparation [37, 41].

## Discussion

Europe has a growing population of ethnic minority groups whose dietary behaviours are potentially of public health concern and the factors driving these behaviours need to be understood. This review identified a broad range of factors and clusters influencing dietary behaviour and identified gaps in the literature to guide future research. The evidence from this review will feed into developing a framework for the study of factors influencing dietary behaviours in ethnic minority populations in Europe.

This review extracted 63 individual factors that were grouped in seven clusters. Two clusters, ‘social and cultural environment’ and ‘food beliefs and perceptions’ had the highest number of factors shown to shape dietary behaviours of ethnic minority populations. These findings corroborate those of earlier reviews on ethnic minority populations [11, 5], in the sense that like other reviews, most factors identified are related to the socio-cultural environment, cultural beliefs and perceptions around food. In our review, factors are clustered in a way that cut across the more traditionally used socio-ecological levels (individual, family, community and society, see for example [51, 52]). The socio-ecological model presents different layers of influence, with an underlying assumption that the layers operate in linearity. Thus community factors are presumed to influence the individual via the family, whereas certain community factors may directly influence dietary choices, bypassing the family. In addition, the socio-ecological model depicts reality as artificially separating individual and social experiences. Clustering factors into systems may provide a more adequate means of depicting interrelationships between the factors. For example, in our analysis, social norms and identity were clustered together but would have been classified as ‘community’ and ‘individual’ factors using the socio-ecological model. By analysing data in this way, we aimed to explore the underlying mechanisms that shape dietary behaviours, in a holistic, systems-based approach. This is in line with recent work that seeks to understand dietary choice as a social practice [53].

Our review identified some factors that have also been shown to influence dietary behaviours of majority populations. These include food price, income [54], social networks [54], time constraints and food availability [55], although these factors are often reported in low income groups [54]. However, many of the factors known to influence diet among majority populations were not identified in our review. For instance, a recent umbrella review among adults [56] of studies conducted in Europe, Australia and North America, identified political environments, food advertising, late-shift work, behavioural regulation and sedentary behaviour as important correlates of dietary behaviour but these factors were absent in our review. This might be due to a bias in studies amongst ethnic minority groups, as there is a tendency by researchers to focus on socio-cultural factors. Factors that were identified as unique to immigrant origin groups included all factors within the ‘migration context’ cluster, and some from other clusters, e.g.; level of acculturation, cultural identity, availability of traditional foods, familiarity with host country foods, competency in host language, perception of host culture and religious prescriptions. While the majority of studies explored ‘differences’ across ethnic groups, this review found a need for research exploring ‘similarities’. This would be useful in adapting mainstream interventions for ethnic minority groups.

It is important to note that although dietary behaviour is generally considered to be associated with SES [54], in our review SES was inconsistently related to dietary behaviour. For instance, a study conducted among adolescents in Oslo [26] reported that SES using a composite measure of parental occupation, mother’s education, employment status and social security status was not associated with diet quality across the study sample of South Asian and African adolescents. In contrast, in other studies [27–29], education was identified to be a determinant of selected food intakes, including fruit and vegetables among elderly Iranian-born residents of Stockholm. The inconsistencies in the relationship between SES and diet corroborates previous studies that have observed differences between SES and metabolic outcomes in different migrant populations [57]. It has been hypothesised that these groups might be in another stage of epidemiological transition, where diet-related NCDs (and risk factors) are still more prevalent (or equally so) in higher SES groups, as was the case among European populations in the 1950s and 60s.

Almost half the studies (44 %) included in this review were focused on South Asians, which is not surprising given that this group forms the largest ethnic minority group in some countries in northern Europe, where most of the studies were conducted [11]. The ‘psychosocial’ cluster of factors seems relevant only to the South Asian population in our review, however, this is a reflection of only one study, therefore, there is insufficient evidence to be able to conclude that these factors are only important to South Asian populations. The findings should be interpreted with caution as not all factors may be applicable to all ethnic minority groups, as they are heterogeneous populations in terms of their acculturation level, ethnicity, socio-demographic status and religion.

Factors that were included in the ‘social and cultural environment’ and ‘food beliefs and perceptions’ clusters appeared to influence dietary behaviours amongst almost all minority groups. Among studies conducted with South Asians (Pakistani, Bangladeshi, Indians) and other migrants from predominantly Muslim countries, religious beliefs and prescriptions were identified as important factors.

### Strengths and limitations of the review

This is the first systematic mapping review that has mapped out factors influencing dietary behaviour among a diverse population of minority groups living in Europe. One difference between this review and others is in the method used in synthesizing the findings. Most reviews have used existing frameworks for this purpose [52, 58]. The approach used in this review has resulted in clustering of factors that transcends existing models, aiming to better capture the complexity of the system of factors influencing dietary behaviour. Another strength of this review was the inclusion of indigenous groups and Eastern Europe migrants to Western Europe in the search strategy, recognising that these groups are potentially disadvantaged and marginalized and may be more vulnerable to NCDs. However, only three studies were identified that reported factors influencing dietary behaviours among the Sami [47, 59, 60] and minority groups from the former Eastern Bloc European countries who commonly migrate to other parts of Europe [28]. We found no studies on Roma populations.

Although migration is a phenomenon that is found in all European countries, most studies were conducted in Northern Europe, and predominantly among populations of South Asian origin. Few studies were conducted among children and older adults. Findings need to be interpreted with caution as factors might differ across age groups. Many studies excluded in the reviewing process focused on describing dietary differences and did not present findings on the factors driving behaviour, which partly accounts for the limited number of relevant studies included in the review. The inclusion of mainly cross-sectional quantitative studies in this review reflects the types of studies available, which also means that we cannot establish causal relationships between the factors identified and dietary behaviours. Whilst there was no limitation for language during the search strategy, our review consists of articles published entirely in English. This could be due to the fact that other relevant articles may not have been indexed in the electronic databases used for this review.

### Implications of the findings

Future research is needed to further deepen our understanding of the interrelationships between identified factors both within and between clusters. In addition, future studies need to make direct comparisons between minority and majority populations to understand differences and commonalities in factors underlying dietary behaviours and food choice. We also recommend studies into a broader range of more ‘mainstream’ factors (as with the majority population). There is also a need for more studies including longitudinal data of factors influencing dietary behaviours across the life course, particularly of young people and older adults among ethnic minority groups. Finally, a gap was identified for studies comparing the drivers of dietary behaviours across a wide range of ethnic minority groups living in different contexts in Europe (including central and southern Europe) and including groups that are under-represented in national surveys such as the Roma, asylum seekers and refugees, which are increasingly relevant groups in Europe.

## Conclusions

This review identified a broad range of factors and clusters of factors potentially influencing dietary behaviour among ethnic minority populations. Gaps in the literature included a need for researchers to explore the underlying mechanisms that shape dietary behaviours, which can be gleaned from more holistic, systems-based studies exploring relationships between factors and clusters. The dominance of studies exploring ‘differences’ between ethnic minority groups and the majority population in terms of the socio-cultural environment and food beliefs suggests a need for research exploring ‘similarities’, that is the relative importance of factors influencing dietary behaviour in the general population in ethnic minority populations. This review shows that the range of factors that influence dietary behaviours among ethnic minority groups is broad. The evidence from this review will feed into the development of a framework for the study of factors driving influencing dietary behaviours in ethnic minority populations in Europe.

## Abbreviations

BMI, body mass index; DEDIPAC-KH, DEterminants of DIet and Physical Activity Knowledge Hub; NCDs, non-communicable diseases; SES, socioeconomic status

## Additional files

Additional file 1: Systematic search strategy in Medline. (DOCX 15 kb) Additional file 2: Table S2. Quality assessment of quantitative studies [9, 10, 27–30, 40, 41, 48, 50, 51, 60–66]. (DOCX 26 kb) Additional file 3: Table S3. Quality assessment of qualitative studies [8, 26, 31–39, 42–45, 49, 67–69]. (DOCX 21 kb)
