# Supplementary material for: Systematic mapping review of the factors influencing dietary behaviour in ethnic minority groups living in Europe: a DEDIPAC study
Source: Int J Behav Nutr Phys Act. 2016 Jul 28;13:85. doi: 10.1186/s12966-016-0412-8 (PMC4964011; doi:10.1186/s12966-016-0412-8)
Supplement: Additional file 1: — Systematic search strategy in Medline. (DOCX 15 kb) [file 12966_2016_412_MOESM1_ESM.docx]

**Additional file 1: Systematic search strategy in Medline**

| **#** | **Searches** | **Results** |
| --- | --- | --- |
| 1 | *Diet/ or *Food Habits/ or Nutritional Status/ or Food Preferences/ or nutrition*.ti,ab. | 236837 |
| 2 | *"Emigrants and Immigrants"/ or *cultural diversity/ or *Minority Groups/ or *"Transients and Migrants"/ or *Ethnic Groups/ or multiculturalism*.mp. or *ethnic minorit*/ or BME.mp. or black minorit*.mp. or ethnic*.mp. or asylum seeker*.mp. or refugee*.mp. or african caribbean*.mp. or *West Indies/ or *Afro-caribbean*/ or *Non-white/ or *Coloured population/ or *Black*/ or *Afric*/ or *Indi*/ or *Caucasian*/ or *Caribbean*/ or *Arab*/ or *Black Afric*/ or *South Asia*/ or *Trinidad/) and Tobago.ti,ab. [mp=title, abstract, original title, name of substance word, subject heading word, keyword heading word, protocol supplementary concept word, , unique identifier] | 86 |
| 3 | ((((((*chinese*/ or *china/ or *irish traveller*/ or gyps*.mp. or *roma/ or *Asian/ or *Pakistan*/ or *banglades*/ or *turk*/ or *iran/ or *Iranian*/ or *irak*/ or *Maghreb*/ or *tunisi*/ or *morocco*/ or *liby*/ or *algeri*/ or *Surinam*/ or *latin American*/ or *cameroo*/ or *equado*/ or *eritre*/ or *chil*/ or *estoni*/ or *latvi*/ or *lithuani*/ or *goergi*/ or *armeni*/ or *azerbaija*/ or *kazakhsta*/ or *ukrain*/ or *belaru*/ or *moldov*/ or *croati*/ or *Czech republic*/ or *pol*/ or *romani*/ or *serbi*/ or *slovaki*/ or *sloveni*/ or *albani*/ or *bulgari*/ or *macedoni/ or *montenegr*/ or *angol*/ or *beliz*/ or *beni*/ or *bhuta*/ or *boliv*/ or *botswan*/ or *brazil*/ or *Burkina faso/ or *burund*/ or *chad/ or *colombi*/ or *cong*/ or *costa ric*/ or *ivoria*/ or *comor*/ or *cub*/ or *White/) and Black Caribbean.mp.) or *white/) and Black African.mp.) or *White/) and Asian.mp.) or *Mixed ethnic background/ or *multiple ethnic background*/ or *Central African Republic/ or djibout*.mp. or domini*.mp. or equado*.mp. or egyp*.mp. or ethiop*.mp. or fij*.mp. or gabo*.mp. or gambit*.mp. or ghan*.mp. or grenad*.mp. or guatemal*.mp. or guine*.mp. or guinea Bissau.mp. or guyan*.mp. or hait*.mp. or hondura*.mp. or hungar*.mp. or indones*.mp. or ira*.mp. or jamaic*.mp. or jord*.mp. or keny*.mp. or kiribat*.mp. or kore*.mp. or koso*.mp. or leban*.mp. or lesot*.mp. or liber*.mp. or macedon*.mp. or madagassc*.mp. or malaw*.mp. or lao [pdr.mp](http://pdr.mp). or malays*.mp. or mald*.mp. or marshall island*.mp. or maurit*.mp. or mexic*.mp. or micronesi*.mp. or moldov*.mp. or mongol*.mp. or mozambi*.mp. or myanma*.mp. or namib*.mp. or nep*.mp. or nicaragu*.mp. or nig*.mp. or nigeri*.mp. or Dominican [republic.mp](http://republic.mp). or el-salvado*.mp. or kyrgyz republic.ti,ab. [mp=title, abstract, original title, name of substance word, subject heading word, keyword heading word, protocol supplementary concept word, rare disease supplementary concept word, unique identifier] | 831415 |
| 4 | (((((pala* or panam*or papua new guinea or paragu* or per* or phillipen* or rwand* or samo* or sao tome) and principe) or Senegal* or Seychelles or sierra leon* or solomon islands or Somali* or south afric* or south suda* or sri lank* or st luc* or st vincen* or suda* or surina* or Swaziland or Syrian arab republic or tajikista* or tanzan* or thail* or timoe-leste or tog* or tong* or turkmensista* or tuval* or ugand* or uzbekist* or vanuat* or venezuel* or vietna* or west bank) and gaza) or yemen or zambi* or zimbabw*).ti,ab. | 8747 |
| 5 | 2 or 3 or 4 | 838745 |
| 6 | Europe/ or *Russia/ or *Ukraine/ or *France/ or *Spain/ or *Sweden/ or *Norway/ or *Germany/ or *Finland/ or *Poland/ or *Italy/ or *Great Britain/ or *Romania/ or *"Republic of Belarus"/ or *Kazakhstan/ or *Greece/ or *Bulgaria/ or *Iceland/ or *Hungary/ or *Portugal/ or *Austria/ or *Czech Republic/ or *Serbia/ or *Ireland/ or *Latvia/ or *Bosnia-Herzegovina/ or *Croatia/ or *Lithuania/ or *Slovakia/ or *Estonia/ or *Denmark/ or *Netherlands/ or *Switzerland/ or *Moldova/ or *Belgium/ or *Albania/ or *"Macedonia (Republic)"/ or *Turkey/ or *Slovenia/ or *Montenegro/ or *cyprus/ or *malta/ or *Azerbaijan/ or *Luxembourg/ or *Georgia/ or *Andorra/ or *Liechtenstein/ or *Monaco/ or *Vatican City/ or *San Marino/ or (Europ or Russia or Ukraine or France or Spain or Sweden or Norway or Germany or (Finland or Poland or Italy or United Kingdom or Great Britain or Romania or Belarus) or (Kazakhstan or Greece or Bulgaria or Iceland or Hungary or Portugal or Austria or Czech Republic or Serbia or Republic of Ireland or Latvia) or (Lithuania or Croatia or Slovakia or Estonia or Denmark or Netherlands or Switzerland or Moldova or Belgium or Albania or Macedonia or Turkey or Slovenia or Montenegro or Cyprus or Azerbaijan or Luxembourg or Georgia or Andorra or Malta or Liechtenstein or San Marino or Monaco or Vatican city) or (Bosnia adj1 Herzegovina)).ti,ab. | 414123 |
| 8 | 1 and 5 and 6 | 451 |
