# Supplementary material for: Systematic mapping review of the factors influencing dietary behaviour in ethnic minority groups living in Europe: a DEDIPAC study
Source: Int J Behav Nutr Phys Act. 2016 Jul 28;13:85. doi: 10.1186/s12966-016-0412-8 (PMC4964011; doi:10.1186/s12966-016-0412-8)
Supplement: Additional file 2: Table S2. — Quality assessment of quantitative studies [9, 10, 27–30, 40, 41, 48, 50, 51, 60–66]. (DOCX 26 kb) [file 12966_2016_412_MOESM2_ESM.docx]

**Additional file 2: Table S2: Quality assessment of quantitative studies**

|  | **Quantitative Scoring Criteria Items** | | | | | | | | | | | | | |  |
| --- | --- | --- | --- | --- | --- | --- | --- | --- | --- | --- | --- | --- | --- | --- | --- |
| **Study** | **1** | **2** | **3** | **4** | **5** | **6** | **7** | **8** | **9** | **10** | **11** | **12** | **13** | **14** | **Summary score** |
| Koochek et al., 2001 [28] | 2 | 2 | 2 | 2 | n/a | n/a | n/a | 2 | 1 | 2 | 2 | 2 | 2 | 2 | 21/22 |
| Volken et al., 2013 [29] | 2 | 2 | 2 | 2 | n/a | n/a | n/a | 2 | 2 | 2 | 2 | 2 | 2 | 2 | 22/22 |
| Edwards et al., 2010 [40] | 2 | 0 | 1 | 2 | n/a | n/a | n/a | 0 | 0 | 2 | 2 | 0 | 2 | 1 | 12/22 |
| Ross 2009 [60] | 2 | 2 | 2 | 2 | n | n | n | 1 | 1 | 1 | 1 | 0 | 2 | 2 | 16/22 |
| Skreblin et al., 2003 [30]. | 2 | 1 | 1 | 2 | n | n | n | 2 | 1 | 1 | 0 | 0 | 2 | 2 | 14/22 |
| Brustad et al., 2008 [48] | 2 | 1 | 2 | 2 | n | n | n | 2 | 1 | 1 | 1 | 0 | 2 | 2 | 16/22 |
| Brustad et al., 2008 [61] | 2 | 2 | 2 | 2 | n/a | n/a | n/a | 2 | 2 | 2 | 2 | 2 | 2 | 2 | 22/22 |
| Kumar et al., 2004 [27] | **2** | **2** | **2** | **2** | na | na | na | 1 | 2 | 2 | 2 | 1 | 1 | 2 | 19/22 |
| Kassam-Khamis et al., 2000 [9] | 1 | 1 | 1 | 0 | na | na | na | 1 | 1 | 1 | 0 | 0 | 2 | 1 | 10/22 |
| Harding et al., 2008 [62] | 2 | 2 | 1 | 2 | na | na | na | 2 | 2 | 2 | 2 | 2 | 2 | 2 | 21/22 |
| Nicolaou et al., 2006 [10] | 2 | 2 | 2 | 2 | na | na | na | 2 | 2 | 2 | 2 | 2 | 2 | 2 | 22/22 |
| Nielsen et al., 2014 [51] | 2 | 2 | 1 | 1 | n | n | n | 1 | 2 | 2 | 1 | 1 | 2 | 2 | 17/22 |
| Carrus G et al., 2009 [50] | 2 | 1 | 1 | 1 | n | n | n | 1 | 1 | 2 | 2 | 1 | 2 | 2 | 16/22 |
| Perez-Cueto, 2009 [41] | 2 | 2 | 1 | 2 | n/a | n/a | n/a | 1 | 2 | 2 | 2 | 2 | 2 | 2 | 20/22 |
| Kjøllesdal et al., 2014 [63] | 2 | 2 | 2 | 1 | 0 | 0 | n/a | 1 | 2 | 2 | 2 | 2 | 2 | 2 | 20/26 |
| Kjollesdal et al., 2010 [64] | 2 | 1 | 1 | 2 | 1 | 1 | 0 | 2 | 2 | 2 | 2 | 0 | 1 | 1 | 18/28 |
| Khunti et al., [65] | 2 | 2 | 2 | 1 | n/a | n/a | n/a | 1 | n/a | 2 | n/a | n/a | 2 | 2 | 14/16 |
| Johansen et al., [66] | 2 | 2 | 1 | 2 | 1 | n/a | n/a | 2 | 2 | 2 | 2 | 2 | 2 | 2 | 22/24 |
