# Supplementary material for: Systematic mapping review of the factors influencing dietary behaviour in ethnic minority groups living in Europe: a DEDIPAC study
Source: Int J Behav Nutr Phys Act. 2016 Jul 28;13:85. doi: 10.1186/s12966-016-0412-8 (PMC4964011; doi:10.1186/s12966-016-0412-8)
Supplement: Additional file 3: Table S3. — Quality assessment of qualitative studies [8, 26, 31–39, 42–45, 49, 67–69]. (DOCX 21 kb) [file 12966_2016_412_MOESM3_ESM.docx]

**Additional file 3: Table S3: Quality assessment of qualitative studies**

| **Qualitative Scoring Criteria Items** |  |  |  |  |  |  |  |  |  |  |  |
| --- | --- | --- | --- | --- | --- | --- | --- | --- | --- | --- | --- |
| **Study** | **1** | **2** | **3** | **4** | **5** | **6** | **7** | **8** | **9** | **10** | **Summary score** |
| Lawrence et al., 2007 [44] | 2 | 2 | 2 | 1 | 1 | 1 | 1 | 0 | 2 | 2 | 14/20 |
| Lawton et al., 2008 [37] | 2 | 2 | 2 | 1 | 2 | 2 | 2 | 0 | 2 | 1 | 16/20 |
| Fargerli et al., 2005 [26] | 2 | 2 | 2 | 0 | 2 | 2 | 2 | 2 | 2 | 2 | 18/20 |
| Garnweidner et al., 2012 [42] | 2 | 1 | 2 | 2 | 2 | 2 | 2 | 2 | 2 | 2 | 19/20 |
| Grace et al., 2008 [39] | 2 | 2 | 2 | 2 | 1 | 2 | 1 | 1 | 1 | 1 | 15/20 |
| Halkier et al., 2011 [67] | 2 | 1 | 2 | 2 | 1 | 1 | 1 | 1 | 1 | 2 | 14/20 |
| Kohinor et al., 2011 [31] | 2 | 2 | 2 | 1 | 2 | 2 | 1 | 1 | 2 | 1 | 16/20 |
| Ahlqvist et al., 2000 [68] | 2 | 2 | 2 | 1 | 2 | 2 | 2 | 2 | 2 | 1 | 18/20 |
| Terrangi et al., 2014 [43] | **2** | **2** | **2** | **1** | **1** | **1** | **1** | **0** | **2** | **1** | 13/20 |
| Jonsson et al., 2002 [32] | 2 | 2 | 2 | 2 | 2 | 2 | 2 | 1 | 1 | 0 | 13/20 |
| Hendriks et al., 2012 [33] | 1 | 1 | 1 | 2 | 2 | 2 | 1 | 0 | 1 | 0 | 11/20 |
| Rawlins et al., 2013 [38] | 2 | 1 | 2 | 1 | 1 | 1 | 1 | 0 | 2 | 2 | 11/20 |
| Tuomainen, 2009 [34]. | 2 | 2 | 2 | 2 | 2 | 1 | 1 | 0 | 2 | 0 | 14/20 |
| Nicolaou et al., 2009 [35] | 2 | 2 | 2 | 2 | 2 | 2 | 1 | 2 | 2 | 2 | 19/20 |
| Nicolaou et al., 2013 [8] | 2 | 1 | 2 | 2 | 1 | 0 | 0 | 0 | 1 | 2 | 11/20 |
| Nicolaou et al., 2012 [45] | 2 | 2 | 2 | 2 | 2 | 2 | 2 | 0 | 2 | 2 | 18/20 |
| Nielsen, 2013 [49] | 2 | 2 | 2 | 2 | 1 | 2 | 2 | 0 | 2 | 1 | 16/20 |
| Jonsson, 2002 [36] | 2 | 2 | 2 | 2 | 2 | 2 | 2 | 1 | 1 | 0 | 16/20 |
| Mellin-Olsen et al., 2005 [69] | 2 | 2 | 2 | 2 | 2 | 1 | 2 | 1 | 2 | 2 | 18/20 |
